# Prevalence and incidence density rates of chronic comorbidity in type 2 diabetes patients: an exploratory cohort study

**DOI:** 10.1186/1741-7015-10-128

**Published:** 2012-10-29

**Authors:** Hilde Luijks, Tjard Schermer, Hans Bor, Chris van Weel, Toine Lagro-Janssen, Marion Biermans, Wim de Grauw

**Affiliations:** 1 Department of Primary and Community Care, Radboud University Nijmegen Medical Centre, PO Box 9101, 6500 HB Nijmegen, the Netherlands

**Keywords:** type 2 diabetes, comorbidity, primary care, prevalence, incidence

## Abstract

**Background:**

Evidence-based diabetes guidelines generally neglect comorbidity, which may interfere with diabetes management. The prevalence of comorbidity described in patients with type 2 diabetes (T2D) shows a wide range depending on the population selected and the comorbid diseases studied. This exploratory study aimed to establish comorbidity rates in an unselected primary-care population of patients with T2D.

**Methods:**

This was a cohort study of 714 adult patients with newly diagnosed T2D within the study period (1985-2007) in a practice-based research network in the Netherlands. The main outcome measures were prevalence and incidence density rates of chronic comorbid diseases and disease clusters. All chronic disease episodes registered in the practice-based research network were considered as comorbidities. We categorised comorbidity into 'concordant' (that is, shared aetiology, risk factors, and management plans with diabetes) and 'discordant' comorbidity. Prevalence and incidence density were assessed for both categories of comorbidity.

**Results:**

The mean observation period was 17.3 years. At the time of diabetes diagnosis, 84.6% of the patients had one or more chronic comorbid disease of 'any type', 70.6% had one or more discordant comorbid disease, and 48.6% and 27.2% had three or more chronic comorbid diseases of 'any type' or of 'discordant only', respectively. A quarter of those without any comorbid disease at the time of their diabetes diagnosis developed at least one comorbid disease in the first year afterwards. Cardiovascular diseases (considered concordant comorbidity) were the most common, but there were also high rates of musculoskeletal and mental disease. Discordant comorbid diseases outnumbered concordant diseases.

**Conclusions:**

We found high prevalence and incidence density rates for both concordant and discordant comorbidity. The latter may interfere with diabetes management, thus future research and clinical practice should take discordant comorbidity in patients with T2D into account.

## Background

Ageing of the population contributes to the increasing prevalence of diabetes [1-4] and of multimorbidity, that is, the co-occurrence of multiple diseases within one person [5]. The prevalence of multimorbidity is estimated at 16 to 58% in adults in primary care or population-based settings [6-9]. Diabetes mellitus (type 2 diabetes; T2D) is a chronic disease with marked effects on mortality and healthcare expenditure [2], and its prevalence in the USA was estimated at 8% in 2010 [10].

When referring to a specific disease such as diabetes as an index condition, any co-occurring conditions are considered comorbidity [5,11]. In primary care, over 40% of patients with diabetes also have comorbidity [12], which is as high as 70 to 95% in selected diabetes cohorts [13,14]. Diabetes treatment may provide lower benefit to patients with diabetes and comorbidity [15,16]. Comorbidity has a negative effect on the quality of life of patients with diabetes [17-20], and substantially increases their healthcare utilization [12,14]. It also negatively influences their self-management and emotional well-being [21].

The number of studies on comorbidity in T2D is limited. Previous studies focused mainly on 'concordant' comorbidity, that is, conditions that share pathogenesis, risk factors, and/or management plans with T2D (for instance, hypertension) [22]. 'Discordant' combinations [22], that is, diseases without shared pathogenesis, risk factors, or management, remain largely unexplored. Diabetes patients with concordant and discordant comorbidity show similarly increased healthcare utilization [12]. Recommendations for clinical approaches to comorbidity in general and of discordant combinations in particular are rarely provided in evidence-based (diabetes) guidelines [23].

Epidemiologic descriptions of both concordant and discordant comorbidity in an unselected T2D population may increase understanding of the heterogeneity of populations with T2D, and may encourage consideration of co-existing discordant comorbid conditions in current T2D management. To date, epidemiological research on comorbidity in T2D has been limited to prevalence estimates from cross-sectional studies only. The frequency and sequence in which comorbid diseases occur may have important implications for aetiology, prognosis, and management [11]. Consequently, it would be useful to assess the prevalence and the incidence of comorbid diseases in patients with T2D.

The aims of this study were to establish the prevalence and types of an extensive range of chronic comorbid diseases in patients with T2D at the time of their diabetes diagnosis, and to establish the incidence density of new chronic comorbid diseases in these patients over time. We limited neither the number nor the types of chronic comorbid diseases to be studied in advance.

## Methods

### Design, setting, and patients

We performed a cohort study in a population of 714 patients with newly diagnosed T2D (patient demographics are shown in Table 1), using morbidity data from all patients with newly diagnosed T2D from the Continuous Morbidity Registration (CMR), a practice-based research network in the Nijmegen region located in the eastern part of the Netherlands. In the Netherlands, all patients are listed with a general practitioner (GP) and receive professional healthcare through this GP. The CMR consists of four general practices, in which the GPs have been recording prospectively all episodes of morbidity for all enlisted patients from 1967 onwards, including diagnoses made by specialists after referral [24]. Diagnoses recorded in the CMR have been shown to have high validity [25,26]. In general, longitudinal data collected in research networks such as the CMR are representative of primary care [27]. The CMR contains each patient's date of birth, gender and socioeconomic status (SES), based on the Dutch Standard Classification of Occupations [28], classified as low, moderate, or high [29]. For many years, the total population in these practices had been relatively stable, at around 12,000 patients, with approximately 80% being adults. From 1998 onwards, the population increased steadily, reaching 14,000 in 2006 [30]. Data from the CMR are representative for distribution of age, gender, and SES in the Netherlands [30,31].

**Table 1:** Characteristics of the 714 patients included in the study

Variables	
Sex, n (%)	
Male	351 (49.2)
Female	363 (50.8)

SES,^1 ^n (%)	
Low	362 (50.7)
Middle	282 (39.5)
High	62 (8.7)
Missing	8 (1.1)

Age at diabetes diagnosis, years, mean (± SD, range)	
Total	63.2 (± 12.8, 21 to 95)^2^
Males	61.9 (± 12.8, 21 to 94)
Females	64.4 (± 12.7, 23 to 95)

Time after diabetes,^3 ^years, mean (± SD)	6.2 (± 4.7)^2^
Males	5.9 (± 4.5)
Females	6.6 (± 4.9)

Time before diabetes,^4 ^years, mean (± SD)	11.1 (± 6.3)

Time in study population, years, mean (± SD)	17.3 (± 6.0)

Year of diagnosis,^5 ^, n (%)	
1985-1989	100 (14.0)
1990-1999	276 (38.7)
2000-2006	338 (47.3)

Reason for follow-up ending, n (%)	
End of study period	462 (64.7)
Deceased	155 (21.7)
Moved/left practice	97 (13.6)

^1^SES, socioeconomic status.

^2^Significant difference for males and females (*P *< 0.05). Non-significant gender differences not shown.

^3^Observation time in the study population after diabetes diagnosis.

^4^Observation time in the study population before diabetes diagnosis.

^5^Year of diagnosis of diabetes, classified into categories corresponding to the latest issues of the Dutch College of General Practitioner's T2D guideline. The first, second and third issues were published in 1989, 1999, and 2006 respectively.

Studies based on CMR data comply with the Code of Conduct for Health Research, which has been approved by the Data Protection Authorities for conformity with the applicable Dutch privacy legislation. For this study, approval of an external ethics committee was not required.

This explorative study period covered the years 1985 to 2007. During this period, the CMR's morbidity classification system was not changed, which enabled us to compare identical diagnoses consistently over time. Our study population had a dynamic composition, that is, observation period from start to end points varied between patients. The observation time for individual patients began with the start of the study period (1 January 1985), including for patients who had already been registered in the CMR database before 1985, or the date of a patient's enrolment as a patient in the CMR, whichever occurred first. The observation period for patients terminated either at the end of our study period (31 December 2006), or with a patient's death or deregistration from the practice, whichever occurred first. We included all adult patients (aged 18 years or over) with T2D. The diabetes diagnosis had to be made within the study period (that is, incident cases) in accordance with universally accepted criteria [32], and was verified in the patient's medical record when the age at the time of diagnosis was less than 45 years. Diabetes care in the CMR practices has been shown to achieve outcomes comparable with those reported under randomized controlled trial conditions [33].

### Comorbidity

We considered all chronic diseases as comorbidities, regardless of whether they occurred before or after the patient's diabetes diagnosis. The CMR distinguishes approximately 500 diagnostic codes (the 'E-list codes'). The GPs label each code as a new or ongoing episode for a known disease. No generally accepted definition of 'chronicity' exists, but frequently used criteria for chronicity include duration, pattern with recurrence or deterioration, and consequences on a patient's life measured by various outcomes [34]. For the current study, we defined chronic conditions as diseases (a) that are persistent (duration of 6 months or longer); (b) from which the patient does not recover; and (c) that require healthcare attention. Those conditions that did not evidently fulfil all three criteria were presented to a panel of eight experienced GPs from the CMR practices, who categorised each condition as chronic, non-chronic or conditionally chronic. We distinguished 'conditionally chronic' diseases as those that can but do not need to have a chronic course, depending on the individual; examples are depression, asthma, and epilepsy. In these cases, the ongoing episodes at patient level defined the presence or absence of chronicity for this individual. When the expert panel unanimously judged a specific disease as chronic, we considered this particular disease as chronic in further analyses. In cases of disagreement between the panel members, the disease was labeled as conditionally chronic. In these cases, ongoing episodes at the patient level defined individual chronicity. All chronic diseases were regarded as cases of comorbidity of T2D. The final list contained 67 chronic and 63 conditionally chronic disorders (see Additional file 1).

Finally, comorbid diseases were classified into clusters, in accordance with the following chapters of *The International Classification of Primary Care *(ICPC)-1: cardiovascular, musculoskeletal, mental, eye, ear, urology, male and female genital system, respiratory, skin, digestive, endocrine and metabolic, neurologic, blood(-forming organs) and lymphatics, and general and unspecified diseases [35]. We also distinguished the subcomponents of infectious diseases and neoplasms (malignancies) as separate clusters. Small and mutually related clusters were combined into one category (see Additional file 2 for the cluster arrangement).

Using T2D as the index disease, we considered all chronic diseases from the cardiovascular cluster as concordant and all other diseases as discordant comorbidity.

### Statistical analysis

We calculated the prevalence of chronic comorbidity at the date of diabetes diagnosis as the number of patients with a specified (cluster of) chronic comorbidity, divided by the total number of patients, and expressed it as a proportion, with 95% confidence interval (CI). A cluster was present if at least one of the chronic diseases within this cluster had been diagnosed in an individual patient.

We also calculated the incidence density rate of chronic comorbidity for the first year before diabetes diagnosis, and for the first year, the first 5 years, and the first 10 years after diabetes diagnosis. We divided the number of new cases of (a cluster of) chronic comorbid diseases within the specified time period by the number of person-years at risk for a diagnosis of that particular comorbidity, and expressed the incidence density rate as the number of new cases per 1,000 patient-years at risk (with 95% CI). Patients who had already developed the particular comorbid disease before the specified period were no longer considered to be at risk, because a chronic disease can be diagnosed only once, and persists subsequently. For incident cases of chronic comorbidity, only the time until diagnosis of this comorbid disease contributed to the number of patient-years.

To analyse the overall burden of comorbidity in our study population, we counted the total number of comorbid chronic diseases and clusters at the time of diabetes diagnosis, and calculated the mean and standard deviation. We also calculated the prevalence and the incidence density for having 'any' chronic comorbidity and for having three or more chronic comorbid diseases or clusters.

We tested patient characteristics for gender differences with the independent *t*-test for continuous variables and the χ^2 ^tests for categorical variables. In all cases, significance was set at *P *≤ 0.05. SPSS software (version 18.0; SPSS Inc., Chicago, IL, USA) supported the analyses.

## Results

### Patient characteristics

The mean ± SD age at diabetes diagnosis was 63.2 ± 12.8 years, and the mean observation time was 17.3 ± 6.0 years. Generally, patients had a longer period before than after diabetes diagnosis within our study period. Patient age showed a normal distribution, whereas time before/after diabetes did not, with over-representation of extreme values (maximum observation time within study period). Women were generally older than men at the time of their diabetes diagnosis and had a longer follow-up, but the total observation time did not differ between women and men. Table 1 shows the patient characteristics; values are shown by gender only for those characteristics with significant gender differences.

### Prevalence of chronic comorbidity at time of diabetes diagnosis

We assessed the prevalence of chronic comorbidity at the time of diabetes diagnosis (Table 2, Table 3). Only 15.4% of the patients did not have chronic comorbidity. Counting discordant diseases only (that is, excluding cardiovascular disease; CVD) showed that 70.6% (95% CI 67.2 to 73.9%) had at least one discordant comorbid disease in addition to T2D.

**Table 2:** Mean ± SD number of (clusters of) chronic comorbid diseases at date of diabetes diagnosis^1,2^

	Single chronic diseases		**Clusters of comorbidity**^3^	
	
	All	**Discordant only**^4^	All	**Discordant only**^4^
**Number**	2.9 ± 2.4	1.7 ± 1.7	2.1 ± 1.5	1.5 ± 1.3

^1^This table describes the mean of chronic comorbid diseases present in our total population (*n *= 714) at the date of diagnosis of type 2 diabetes.

^2^Data are displayed both for the total count of single comorbid diseases and for the number of clusters of chronic comorbid diseases. We also distinguished 'any type' of comorbid diseases and 'discordant diseases only'.

^3^Clusters: comorbid diseases were classified into clusters, following *The International Classification of Primary Care *(ICPC)-1 chapters.

^4^Discordant: without shared pathogenesis, risk factors, or management.

**Table 3:** Prevalence and incidence density of chronic comorbidity: overall, before, at time of, and after diabetes diagnosis (DD)^1,2^

**Time**	**Extent of chronic comorbidity**						
	
		**≥ 1 chronic comorbid disease**		**≥ 3 chronic comorbid diseases**		**≥ 3 clusters**^3^	
		
		**All**	**Discordant**^4 ^**only**	**All**	**Discordant**^4 ^**only**	**All**	**Discordant**^4 ^**only**
	
Before diabetes	ID, year before DD^5^	259.3 (169.4 to 349.1)	106.2 (61.8 to 150.6)	101.9 (69.5 to 134.3)	42.9 (25.0 to 60.8)	78.8 (52.7 to 104.9)	45.4 (27.6 to 63.3)
	Prevalence^6 ^at DD, % (95% CI)	84.6 (81.8 to 87.1)	70.6 (67.2 to 73.9)	48.6 (44.9 to 52.3)	27.2 (24.0 to 30.5)	38.0 (34.5 to 41.6)	22.0 (19.1 to 25.1)
	
After diabetes	ID, first year after DD^5^	263.7 (160.3 to 367.0)	88.3 (46.3 to 130.2)	80.9 (50.4 to 111.4)	38.9 (21.4 to 56.4)	48.3 (27.1 to 69.5)	30.4 (15.5 to 45.4)
	ID, first 5 years after DD^5^	160.2 (116.2 to 204.2)	83.9 (62.3 to 105.5)	91.2 (74.1 to 108.3)	45.1 (35.3 to 54.8)	59.5 (47.3 to 71.6)	36.3 (27.9 to 44.6)
	ID, first 10 years after DD^5^	142.5 (107.0 to 177.9)	84.5 (66.0 to 103.0)	84.8 (70.6 to 99.1)	47.7 (39.2 to 56.1)	62.5 (51.9 to 73.1)	37.6 (30.4 to 44.7)

Abbreviations: ID, incidence density.

^1^This table displays data on the presence and occurrence of one (the first) and of three (the third) chronic comorbid disease(s), and on the presence and occurrence of three (the third) cluster(s) of chronic comorbid disease(s) respectively.

^2^Confidence intervals (CIs) for proportions were calculated with the Mid-P exact test. CIs for person time were calculated as a normal approximation to the Poisson interval.

^3^Clusters: comorbid diseases were classified into clusters, following *The International Classification of Primary Care *(ICPC)-1 chapters.

^4^Discordant: without shared pathogenesis, risk factors or management.

^5^Incidence density displays the number of new occurrences of the specified number of chronic comorbid diseases or clusters of comorbidity, divided by the number of person-years at risk within the specified period. Expressed as number of new cases per 1,000 patient-years at risk (95% CI).

^6^Prevalence at diabetes date displays the proportion of patients in whom (at least) the specified number of chronic comorbid diseases, or clusters of comorbidity, had been diagnosed at this date.

Having three or more chronic comorbid diseases when T2D was diagnosed was not uncommon: approximately half (48.6%; 95% CI 44.9 to 52.3) of the population had at least one chronic comorbid disease, and approximately a quarter (27.2%; 95% CI 24.0 to 30.5) had three or more discordant chronic comorbid diseases. From the prevalence data of diseases from different clusters (Table 3), it follows that this was often a heterogeneous mix of diseases.

CVDs were the most prevalent comorbid diseases at the time of diabetes diagnosis: 64.0% (95% CI 60.4 to 67.5) (Table 4). Musculoskeletal and mental diseases were also very common. There was a high prevalence of chronic functional somatic symptoms [36] and deafness as single diseases. Table 5 shows data on the most common chronic comorbid diseases from every cluster.

**Table 4:** Prevalence and incidence density of clusters of chronic comorbidity; before, at time of, and after diabetes diagnosis (DD)^1^

	Before diabetes	Date of DD	After diabetes		
	
**Disease cluster**^2^	**ID year before DD**^3^	**Prevalence at DD, % (95% CI)**^4^	**ID first year after DD**^3^	**ID first 5 years after DD**^3^	**ID first 10 years after DD**^3^
Cardiovascular	176.9 (127.9 to 225.9)	64.0 (60.4 to 67.5)	122.3 (77.0 to 167.6)	105.5 (82.9 to 128.0)	101.0 (82.1 to 119.9)

Musculoskeletal	31.3 (15.5 to 47.1)	31.1 (27.8 to 34.6)	21.6 (8.2 to 35.0)	27.9 (20.1 to 35.8)	31.6 (24.6 to 38.6)

Mental	15.2 (4.7 to 25.8)	24.1 (21.1 to 27.3)	5.8 (0.0 to 12.4)	10.4 (5.9 to 14.8)	12.6 (8.5 to 16.7)

Eye and ear	29.6 (15.1 to 44.0)	22.7 (19.7 to 25.9)	46.9 (28.1 to 65.6)	45.3 (35.7 to 54.9)	42.8 (34.9 to 50.7)

Urogenital (male and female)	23.7 (11.3 to 36.1)	15.4 (12.9 to 18.2)	17.6 (6.7 to 28.5)	16.9 (11.5 to 22.4)	15.5 (11.2 to 19.8)

Urogenital^5^	26.7 (8.2 to 45.2)	13.4 (10.1 to 17.3)	14.1 (0.3 to 27.9)	12.8 (6.1 to 19.5)	14.1 (8.2 to 20.0)

Urogenital^6^	20.6 (4.1 to 37.0)	17.4 (13.7 to 21.5)	21.0 (4.2 to 37.8)	21.0 (12.4 to 29.6)	16.9 (10.5 to 23.2)

Respiratory	6.8 (0.1 to 13.4)	14.1 (11.7 to 16.9)	3.4 (0.0 to 8.2)	4.3 (1.6 to 6.9)	3.8 (1.7 to 5.8)

Skin	8.0 (1.0 to 15.1)	9.9 (7.9 to 12.3)	1.6 (0.0 to 4.8)	3.8 (1.3 to 6.3)	3.1 (1.3 to 5.0)

Digestive	3.2 (0.0 to 7.5)	8.5 (6.7 to 10.8)	3.2 (0.0 to 7.7)	3.7 (1.3 to 6.1)	4.0 (1.9 to 6.1)

Endocrine and metabolic	9.4 (1.9 to 17.0)	8.4 (6.5 to 10.6)	4.8 (0.0 to 10.3)	6.7 (3.4 to 9.9)	5.7 (3.2 to 8.2)

Malignancies	14.0 (4.8 to 23.1)	7.3 (5.5 to 9.4)	14.4 (5.0 to 23.7)	15.9 (10.9 to 20.9)	17.5 (13.2 to 21.9)

Neurologic	1.5 (0.0 to 4.4)	3.4 (2.2 to 4.9)	1.5 (0.0 to 4.5)	2.0 (0.2 to 3.7)	1.9 (0.5 to 3.3)

Blood and lymphatics	4.4 (0.0 to 9.3)	0.4 (0.1 to 1.1)	0.0	1.5 (0.0 to 3.0)	1.6 (0.3 to 2.8)

Infectious	0.0	0.3 (0.0 to 0.9)	0.0	0.0	0.0

Abbreviations: ID, incidence density.

^1^Confidence intervals (CIs) for proportions were calculated with the Mid-P exact test. CIs for person time were calculated as normal approximation to the Poisson interval. Values smaller than 0.05 have been truncated to 0.0.

^2^Clusters: comorbid diseases were classified into clusters, after *The International Classification of Primary Care *(ICPC)-1 chapters. Sorted by decreasing prevalence at date of DD. The cluster 'general/unspecified' was removed because it contained only one case.

^3^Incidence density in a specified time period was calculated as the number of new cases of one or more diseases within a cluster of comorbidity, divided by the number of person-years at risk for a diagnosis of the particular cluster. Expressed as number of new cases per 1,000 patient-years at risk (95% CI).

^4^Prevalence at diabetes date was calculated as the number of patients with comorbidity in the cluster of interest at date of DD, divided by the total number of patients (n = 714).

^5^Prevalence and incidence density displayed only for men in the study population.

^6^Prevalence and incidence density displayed only for women in the study population.

**Table 5:** Prevalence and incidence density of chronic comorbid diseases: before, at time of, and after diabetes diagnosis (DD)^1^

**Chronic disease**^2^	Before diabetes	Date of DD	After diabetes		
	
	**ID**,^3 ^**year before DD**	**Prevalence**^4 ^**at DD, % (95% CI)**	**ID**^3^,**first year after DD**	**ID**^3^,**first 5 years after DD**	**ID**^3^, **first 10 years after DD**
Hypertension	75.2 (49.9 to 100.5)	38.4 (34.9 to 42.0)	56.8 (33.6 to 80.0)	42.6 (32.1 to 53.0)	40.0 (31.5 to 48.5)

Varicose veins; venous insufficiency	16.5 (5.7 to 27.3)	21.4 (18.5 to 24.6)	13.3 (3.5 to 23.2)	12.5 (7.6 to 17.4)	10.8 (7.0 to 14.6)

Angina pectoris	13.1 (4.0 to 22.2)	12.3 (10.1 to 14.9)	13.6 (4.2 to 22.9)	14.4 (9.5 to 19.3)	15.1 (10.9 to 19.3)

Atrial fibrillation/flutter	18.9 (8.2 to 29.7)	8.7 (6.8 to 10.9)	8.1 (1.0 to 15.2)	11.2 (7.0 to 15.5)	12.3 (8.6 to 16.0)

Myocardial infarction	17.2 (7.0 to 27.3)	8.5 (6.7 to 10.8)	4.8 (0.0 to 10.3)	7.8 (4.3 to 11.3)	9.6 (6.4 to 12.8)

(Congestive) heart failure	15.6 (5.9 to 25.3)	8.0 (6.2 to 10.2)	16.1 (6.1 to 26.1)	12.7 (8.3 to 17.2)	16.2 (12.0 to 20.4)

CVA	15.1 (5.7 to 24.5)	4.9 (3.5 to 6.7)	12.5 (3.8 to 21.1)	11.2 (7.1 to 15.4)	14.7 (10.7 to 18.6)

Intermittent claudication	9.0 (1.8 to 16.2)	4.3 (3.0 to 6.0)	4.6 (0.0 to 9.9)	4.3 (1.8 to 6.9)	3.5 (1.6 to 5.4)

TIA	8.9 (1.8 to 16.0)	2.9 (1.9 to 4.4)	4.6 (0.0 to 9.8)	5.5 (2.6 to 8.3)	5.9 (3.4 to 8.4)

Heart valve disease	6.0 (0.1 to 11.8)	2.9 (1.9 to 4.4)	7.6 (0.9 to 14.3)	5.8 (2.9 to 8.7)	5.6 (3.2 to 8.0)

Osteoarthritis, knee	14.7 (5.1 to 24.3)	12.0 (9.8 to 14.6)	6.7 (0.1 to 13.3)	13.9 (9.1 to 18.8)	13.3 (9.4 to 17.2)

Osteoarthritis, hip	7.9 (1.0 to 14.9)	8.7 (6.8 to 10.9)	1.6 (0.0 to 4.8)	3.7 (1.3 to 6.1)	5.4 (3.0 to 7.8)

Osteoarthritis, other	7.9 (1.0 to 14.8)	8.4 (6.5 to 10.6)	9.7 (1.9 to 17.5)	8.4 (4.7 to 12.0)	8.1 (5.1 to 11.1)

Osteoarthritis, cervical spine	6.2 (0.1 to 12.3)	7.1 (5.4 to 9.2)	4.8 (0.0 to 10.2)	2.4 (0.5 to 4.4)	2.5 (0.9 to 4.1)

Lumbar osteoarthritis	4.7 (0.0 to 9.9)	7.0 (5.3 to 9.1)	8.0 (1.0 to 14.9)	5.3 (2.4 to 8.2)	4.5 (2.3 to 6.8)

Rheumatoid arthritis; ankylosing spondylarthritis	0.0	1.4 (0.7 to 2.5)	0.0	0.4 (0.0 to 1.1)	1.0 (0.0 to 2.1)

(Chronic) functional somatic symptoms	5.4 (0.0 to 11.5)	19.2 (16.4 to 22.2)	3.7 (0.0 to 8.8)	3.8 (1.2 to 6.4)	3.5 (1.4 to 5.6)

Depression	3.0 (0.0 to 7.1)	2.5 (1.5 to 3.9)	0.0	1.2 (0.0 to 2.5)	1.3 (0.2 to 2.5)

Alzheimer's disease	7.3 (0.9 to 13.8)	1.5 (0.8 to 2.7)	3.0 (0.0 to 7.2)	5.0 (2.3 to 7.7)	7.1 (4.4 to 9.8)

Mental retardation	0.0	1.5 (0.8 to 2.7)	0.0	0.0	0.0

Deafness	16.6 (6.3 to 26.9)	13.6 (11.2 to 16.3)	13.7 (4.2 to 23.2)	15.5 (10.3 to 20.6)	13.7 (9.7 to 17.7)

Cataract	17.1 (7.0 to 27.2)	7.8 (6.0 to 10.0)	42.8 (26.3 to 59.2)	34.0 (26.4 to 41.6)	33.6 (27.2 to 40.0)

Glaucoma	3.0 (0.0 to 7.1)	2.9 (1.9 to 4.4)	3.0 (0.0 to 7.3)	3.1 (1.0 to 5.3)	2.4 (0.8 to 4.0)

Urinary incontinence^5^	13.1 (0.3 to 26.0)	13.2 (10.0 to 17.0)	16.6 (2.1 to 31.2)	17.3 (9.7 to 24.8)	14.7 (8.9 to 20.4)

Prostatic hyperplasia/hypertrophy^6^	19.3 (3.9 to 34.8)	9.7 (6.9 to 13.1)	3.4 (0.0 to 10.0)	8.7 (3.3 to 14.1)	10.4 (5.5 to 15.4)

Uterine fibroid^5^	0.0	2.2 (1.0 to 4.1)	0.0	0.7 (0.0 to 2.2)	0.5 (0.0 to 1.7)

Urinary incontinence^6^	9.0 (0.0 to 19.1)	2.0 (0.9 to 3.9)	9.4 (0.0 to 19.9)	5.7 (1.5 to 9.9)	5.1 (1.8 to 8.4)

COPD	3.3 (0.0 to 7.8)	11.2 (9.0 to 13.7)	1.7 (0.0 to 4.9)	3.7 (1.3 to 6.2)	3.4 (1.5 to 5.3)

Asthma	3.0 (0.0 to 7.1)	3.1 (2.0 to 4.6)	3.0 (0.0 to 7.3)	1.2 (0.0 to 2.5)	0.8 (0.0 to 1.7)

Psoriasis	4.6 (0.0 to 9.8)	5.6 (4.1 to 7.5)	1.6 (0.0 to 4.6)	2.0 (0.2 to 3.7)	1.6 (0.0 to 1.7)

Diaphragmatic hernia	1.5 (0.0 to 4.4)	2.5 (1.5 to 3.9)	0.0	0.0	0.3 (0.0 to 0.8)

Colonic diverticula; diverticulitis	1.5 (0.0 to 4.4)	2.0 (1.1 to 3.2)	3.0 (0.0 to 7.2)	2.3 (0.5 to 4.2)	2.1 (0.6 to 3.6)

Gout	4.5 (0.0 to 9.7)	4.1 (2.8 to 5.7)	0.0	3.5 (1.2 to 5.9)	3.0 (1.2 to 4.7)

Hypothyroidism	4.4 (0.0 to 9.5)	2.5 (1.5 to 3.9)	1.5 (0.0 to 4.5)	1.6 (0.0 to 3.1)	1.6 (0.3 to 2.9)

Hyperthyroidism	0.0	2.2 (1.3 to 3.5)	3.0 (0.0 to 7.2)	1.2 (0.0 to 2.5)	0.8 (0.0 to 1.7)

Breast cancer^5^	2.9 (0.0 to 8.7)	2.8 (1.4 to 4.9)	8.9 (0.0 to 18.9)	7.5 (2.8 to 12.1)	7.1 (3.4 to 10.9)

Prostate cancer^6^	0.0	1.7 (0.7 to 3.5)	6.2 (0.0 to 14.8)	2.4 (0.0 to 5.1)	3.9 (1.0 to 6.8)

Endometrial cancer^5^	0.0	1.4 (0.5 to 3.0)	2.9 (0.0 to 8.6)	0.7 (0.0 to 2.2)	0.5 (0.0 to 1.5)

Skin cancer	2.9 (0.0 to 7.0)	0.7 (0.3 to 1.5)	0.0	1.1 (0.0 to 2.4)	1.8 (0.5 to 3.2)

Colon cancer	0.0	0.6 (0.2 to 1.3)	0.0	1.5 (0.0 to 3.0)	2.1 (0.6 to 3.5)

Lung/bronchial cancer	1.5 (0.0 to 4.3)	0.3 (0.0 to 0.9)	3.0 (0.0 to 7.1)	1.5 (0.0 to 3.0)	1.8 (0.5 to 3.1)

Migraine	0.0	2.0 (1.1 to 3.2)	1.5 (0.0 to 4.5)	0.8 (0.0 to 1.8)	1.1 (0.0 to 2.1)

Abbreviations: DD, date of diabetes diagnosis; COPD, chronic obstructive pulmonary disease; CVA, cerebrovascular accident; ID, incidence density; TIA, transient ischemic attack.

^1^Confidence intervals for proportions were calculated with the Mid-P exact test. Confidence intervals for person time were calculated as a normal approximation to the Poisson interval. Values smaller than 0.0 have been truncated to 0.0.

^2^Diseases sorted by cluster, in decreasing prevalence at the date of DD diabetes date, and within the particular cluster by decreasing prevalence at date of DD.

^3^Incidence density in a specified time period was calculated as the number of new cases of comorbidity, divided by the number of person-years at risk for a diagnosis of the particular comorbidity. Expressed as number of new cases per 1,000 patient-years at risk (95% CI).

^4^Prevalence at diabetes date was calculated as the number of patients with the comorbidity of interest at the date of DD, divided by the total number of patients (n = 714).

^5^Prevalence and incidence displayed only for women in the study population.

^6^Prevalence and incidence displayed only for men in the study population.

Prevalent chronic psychosis, obsessive compulsive disorder, phobia, schizophrenia, dementia, mental retardation, or Down's syndrome were combined as a heterogeneous group of chronic diseases affecting patients' mental states, which were found to affect 3.8% of the total population at time of diabetes diagnosis.

### Chronic comorbidity before diabetes diagnosis

The incidence density rate of any chronic comorbidity (both concordant and discordant) in the year before diabetes diagnosis was very high (Table 3). In general, comorbid disease clusters with high prevalence rates at diabetes diagnosis also had high incidence density rates in the year before diabetes diagnosis (Table 4). For some diseases and clusters, the incidence density rate in the year before diabetes was particularly high compared with the prevalence rate at this time, and also with the incidence density rate after diabetes diagnosis. Examples are CVD (especially myocardial infarction) and male urogenital diseases (Table 4, Table 5).

### Chronic comorbidity after diabetes diagnosis

In the years after diabetes diagnosis, the incidence density rate of chronic comorbidity remained high. A quarter of those without any chronic comorbid disease at the time of diabetes diagnosis developed at least one comorbid disease in the first subsequent year (263.7 new cases per 1,000 patient-years at risk, 95% CI 160.3 to 367.0) (Table 3).

Eye and ear diseases (cataract in particular) had a high incidence density rate after diabetes diagnosis as compared with the year before diagnosis, and also compared with the prevalence rate at diabetes diagnosis: 46.9 per 1,000 patient-years at risk during the first year. Skin diseases and respiratory and endocrine diseases had a lower incidence density rate after diabetes diagnosis than before. The incidence density rate of mental diseases was particularly low in the first year after diabetes diagnosis, with no new cases of chronic depression the first year after diabetes diagnosis (Table 4, Table 5).

## Discussion

### Principal findings

In this study, we established the prevalence and incidence of comorbidity in patients with T2D. We found that 84.6% of the patients with newly diagnosed T2D in a primary-care population had at least one chronic comorbid disease at the time of their diagnosis, and both concordant and discordant comorbidity were common. Incidence density rates after diabetes diagnosis showed that rates of chronic comorbidity further increased after diabetes onset. This study clearly showed the heterogeneity of this primary-care population with T2D in terms of comorbidity.

### Relation to other studies

The prevalence of comorbidity in patients with T2D in this study was similar to [14,37] or higher than [12,13] those of previous studies. The number of comorbid diseases considered in a study contributes to any prevalence estimate [7,38], and our work had the largest number. The relatively high prevalence of comorbidity we found is more pronounced when one considers that we investigated a primary-care population including all adult T2D patients, as opposed to studies that included only patients over 65 years of age [14,37] or those requiring inpatient diabetes treatment [13]. Patients with discordant comorbidity outnumbered those with concordant comorbidity, a finding similar to earlier CMR-based research on comorbidity in patients with heart failure as the index disease [39]. Diabetes is not necessarily causally related to additional diseases, but co-existing chronic diseases may interfere with diabetes management in several ways [14-20]. These results encourage us to reflect on the general lack of attention to (discordant) comorbidity in evidence-based diabetes guidelines [23].

Patients with comorbidity may prioritize one condition over another, and experience overwhelming effects of an individual disease [21,40]. A recent study showed that physician-experienced complexity of patients with diabetes increased with prevalent discordant comorbidity, but not with concordant comorbidity, implying that improvement in diabetes management could be made merely by focusing on patient-centred rather than disease-specific interventions [41]. Patient-centred management is exactly what GPs prioritize in the management of multimorbidity [42]; however, the current tendency is to incentivise disease-specific instead of holistic care, thereby counteracting patient-centred approaches [43-46]. The extent of chronic comorbidity in patients with T2D, as shown in the current study, urges an approach of complementing disease-specific strategies with a personalized, generalist approach for the management of patients with multimorbidity [6,42].

### Strengths and limitations of the study

To our knowledge, this study is the first to describe the development of chronic comorbidity over time in patients with T2D. We were able to identify comorbidity diagnosed before diabetes diagnosis. Selection of patients with a diagnosis of T2D and all comorbidity data were based on the most reliable source, that is, physician diagnoses, rather than patient self-report [17,37] or extraction of medication prescriptions [47].

Diabetes in this study served as an example of a common chronic disease with standardized management plans. The objectives of this exploratory study were to establish the prevalence rates of a range of chronic comorbid conditions and their development over time. Given the nature of the CMR database, comparing comorbidity data in our diabetes population with a control group with another index disease, such as osteoarthritis, would have been possible. However, this would have distracted from the intended epidemiologic description of chronic comorbidity in T2D patients. This study did not aim to quantify the comorbidity rate in patients with T2D compared with patients with other chronic diseases, or to compare the rates within specific subgroups of patients with T2D or at different time periods within the study. Considering the large number of comorbid conditions and clusters studied, such comparisons would have resulted in numerous statistically significant differences or interactions of uncertain clinical relevance. Instead, the current epidemiologic description may lead to more detailed exploration of specific conditions or subgroups for future research.

The particular strengths of the study are that the diabetes population we studied was unselected, and that we did not restrict comorbidity only to prevalent or concordant chronic diseases. Our data reflect the total burden of chronic comorbidity in patients with T2D in general.

Currently, no universally accepted definition of 'chronic diseases' is available. Within any definition, personalization of the concept of chronicity to the individual patient level is preferred, although often not attained [34,48]. An Australian primary-care code set applied the same criteria for chronicity as we did [34]. However, by adding the distinction of conditional chronicity based on physician-assigned ongoing episodes, we were able to personalize chronicity in our analyses. For diseases from which patients may recover (for example, depression), or for diseases with either episodic or chronic courses (for example, asthma, gout), we consider our classification comes closer to the correct description of chronicity than would a list with invariable chronic diseases.

### Comments on specific comorbid diseases

Concordant comorbidity (that is, CVDs) showed the highest prevalence and incidence density rates. Although this cluster contained a large number of diseases, the main explanation for the high rate is the concordance with T2D. Care-related factors will have added to this finding. For instance, a GP will be more attentive for T2D in a patient who has had a myocardial infarction. The suggestion that presence of a disease enhances attention for other diseases [49,50] might be particularly the case for concordant combinations.

For discordant combinations also, care-dependent factors might contribute to the high rates of comorbidity. There was an evidently increased incidence of cataract in the year after diabetes diagnosis. The reason for this may be that screening for diabetic retinopathy resulted in earlier diagnosis of, or otherwise unobserved cataract diagnoses. Moreover, people might not raise certain issues until they visit their doctor for other health problems; such restraints can contribute to a higher incidence of conditions such as incontinence in the first years after diabetes diagnosis. These examples illustrate that despite the high rate of comorbidity reported, our results may still be an underestimation, as the comorbidity data refer to disease episodes truly experienced by patients and presented to their GP.

Musculoskeletal diseases have an antagonistic effect on physical exercise, which is part of the recommended treatment for diabetes [11]. Around 30% (3/10) patients with T2D had musculoskeletal disease at time of diabetes diagnosis, and of those unaffected, an additional 32 new cases per 1,000 patient-years at risk followed during the next 10 years. These are substantial figures, which are higher than chronic musculoskeletal diseases in the overall CMR population [30], and these cases are likely to interfere with diabetes management.

Diabetes treatment focuses on prevention of complications [51]. The presence of a malignancy may overshadow the importance of co-existing diabetes, and thus treatment priorities may alter. Dutch researchers found that patients with diabetes who had cancer received less aggressive cancer treatment than those without diabetes [52].

Parallel to the reluctance of GPs to prescribe interventions for depression in patients with comorbidity [53], Dutch GPs might be conservative in 'adding' a chronic mental disease diagnosis after a diagnosis of diabetes. Including less prevalent diseases in our study enabled us to localize a heterogeneous group of patients with chronic comorbidity who possibly have difficulties in self-managing their diabetes. One in 25 patients had chronic psychosis, obsessive compulsive disorder, phobia, schizophrenia, dementia, mental retardation, or Down's syndrome when diagnosed with diabetes. A 'standard' approach to diabetes would often not respond to these patients' abilities or needs.

## Conclusions

This study illustrated the complexity of the T2D population under GP care, in terms of chronic comorbidity. We have shown that the 'straightforward' patient with T2D without (discordant) comorbidity is relatively rare. Management of diabetes demands management of comorbidity, including discordant diseases. Clinical guidelines have an important role in diabetes management, but their external validity may be questioned by taking comorbidity into consideration [23,43,44]. A patient-centred approach can be of added value in the management of patients with diabetes with chronic comorbidity.

In conclusion, this study provides new knowledge on the epidemiology of chronic comorbidity in T2D. We hope it will inform ongoing research in this area, and is taken into account in diabetes management.

## Abbreviations

CMR: Continuous Morbidity Registration; CVD: cardiovascular disease; GP: general practitioner; T2D: type 2 diabetes.

## Competing interests

No competing interests are reported. HL has received a research grant from SBOH, Foundation for Family Medicine Residency Training in the Netherlands. There was no specific funding for the presented work.

## Authors' contributions

HL, TS, and MB developed the concept of the study and its design, with contributions from WdG, HB, TLJ, and CvW. Acquisition of data was performed by HL and HB, and drafting of the manuscript by HL and TS. The statistical analyses were performed by HL, HB, TS, and MB. HB, CvW, TLJ, MB, and WdG critically revised the manuscript for important intellectual content. WdG, CvW and TLJ supervised the study. All authors have read and approved the manuscript for publication. The corresponding author had full access to all the data in the study, and takes responsibility for the integrity of the data and the accuracy of the data analysis.

## Authors' information

HL is a GP resident and PhD candidate; TS is a senior researcher; HB is a senior researcher and statistician; CvW is a GP and professor of primary care; TLJ is a GP and professor of primary care; MB is a senior researcher and epidemiologist; WdG is a GP and senior researcher.

## Pre-publication history

The pre-publication history for this paper can be accessed here:

http://www.biomedcentral.com/1741-7015/10/128/prepub

## Supplementary Material

Additional file 1 **Comorbidity**. Click here for file

Additional file 2 **Clusters of comorbidity**. Click here for file
