# Supplementary material for: Prevalence and incidence density rates of chronic comorbidity in type 2 diabetes patients: an exploratory cohort study
Source: BMC Med. 2012 Oct 29;10:128. doi: 10.1186/1741-7015-10-128 (PMC3523042; doi:10.1186/1741-7015-10-128)
Supplement: Additional file 1 — Comorbidity. [file 1741-7015-10-128-S1.DOC]

**Additional file 1: Comorbidity**

Chronic diseases included as comorbidity

**Chronic diseases** (total number: 67)

| **E-list code** | **Disease** | **ICPC equivalent** |
| --- | --- | --- |
| 0044 | HIV; AIDS | B90 |
| 0500 | Cancer of the mouth / pharynx | D77.02, D77.03, R85 |
| 0510 | Oesophageal cancer | D77.01 |
| 0520 | Cancer of the stomach | D74 |
| 0530 | Colon cancer | D75 |
| 0540 | Rectal cancer | D75 |
| 0550 | Pancreatic cancer | D76 |
| 0560 | Laryngeal / throat cancer | R85 |
| 0570 | Lung / bronchial cancer | R84 |
| 0580 | Breast cancer | X76 |
| 0590 | Uterine cervical cancer | X75 |
| 0600 | Endometrial cancer | X77.01 |
| 0610 | Prostate cancer | Y77 |
| 0620 | Bladder cancer | U76 |
| 0632 | Ovarian cancer | X77.02 |
| 0639 | Genitourinary cancer, other | U75, U77, X77, Y78 |
| 0650 | Brain cancer / tumour | N74 |
| 0660 | Hodgkin disease | B72.01 |
| 0670 | Leukaemia | B73 |
| 0681 | Lymphoma / multiple myeloma | B74.01 |
| 0682 | Metastases; unknown origin | A79 |
| 0689 | Carcinoma, other | D77.04, L71, T71, W72 |
| 0640 + 0641 + 0642 + 0649 | Skin cancer | S77 |
| 0890 | Hypothyroidism | T86 |
| 1101 | Pernicious anaemia | B81.02 |
| 1141 | Polycythaemia | B75 |
| 1142 | Haemophilia | B83.01 |
| 1192 | Haemolytic anaemia, congenital | B78 |
| 1250 | Schizophrenia | P72 |
| 1270 | Alzheimer’s disease | P70 |
| 1380 | Personality disorder | P80 |
| 1401 + 1402 | Mental retardation | P85 |
| 1403 | Child development disorders, pervasive | P99 |
| 1551 | TIA (transient ischemic attack) | K89 |
| 1559 | CVA (cerebrovascular accident) | K90 |
| 1560 | MS (multiple sclerosis) | N86 |
| 1570 | Parkinson’s disease | N87.01 |
| 1800 | Glaucoma | F93 |
| 1811 | Blindness / amblyopia | F94 |
| 1841 | Cholesteatoma | H74.03 |
| 1880 | Otosclerosis | H83 |
| 1890 | Deafness | H84, H86 |
| 2080 | Heart valve disease | K83 |
| 2090 | Heart valve disease *(rheumatic)* | K71.02 |
| 2110 | Myocardial infarction | K75 |
| 2120 | Angina pectoris | K74 |
| 2131 | (Congestive) heart failure | K77 |
| 2132 | Atrial fibrillation / flutter | K78 |
| 2133 | Pulmonary heart disease | K82 |
| 2180 + 2181 + 2189 | Hypertension | K86, K87 |
| 2231 | Intermittent claudication | K92.01 |
| 2480 | COPD (chronic obstructive pulmonary disease) | R95 |
| 2530 | Pneumoconiosis | R99.06 |
| 2540 | Bronchiectasis | R91.02 |
| 2852 | Crohn’s disease; ulcerative colitis | D94 |
| 2881 | Hepatic cirrhosis | D97 |
| 3820 | Psoriasis | S91 |
| 4050 + 4051 + 4052 | Rheumatoid arthritis; ankylosing spondylarthritis | L88.01, L88.02 |
| 4061 | Osteoarthritis, hip | L89 |
| 4062 | Osteoarthritis, knee | L90 |
| 4063 | Lumbar osteoarthritis | L84 |
| 4064 | Osteoarthritis, cervical spine | L84.01 |
| 4069 | Osteoarthritis, other | L91 |
| 4154 | Osteoporosis | L95.02 |
| 4300 | Multiple congenital abnormalities | A90 |
| 4310 | Spinal dysraphism | N85.01 |
| 4389 | Down syndrome / other specified congenital abnormalities | A90(.01) |

**Conditionally chronic diseases*** (total number: 63)

| **Code** | **Disorder** | **ICPC equivalent** |
| --- | --- | --- |
| 0010 | Pulmonary tuberculosis | R70 (ex. A70) |
| 0020 | Tuberculosis, *other organs* | A70 (ex. R70) |
| 0030 | Syphilis | X70 / Y70 |
| 0162 | Hepatitis B | D72.02 |
| 0164 | Hepatitis C | D72.03 |
| 0169 | Hepatitis | D72 |
| 0460 | Sarcoidosis | R83.02 |
| 0470 | Lyme disease | A78.05 |
| 0631 | Testis cancer | Y78.02 |
| 0710 | Uterine fibroid | X78.01 |
| 0821 | Neoplasm malignant / benign | S80 |
| 0860 | Asthma | R96 |
| 0880 | Hyperthyroidism | T85 |
| 0930 | Gout | T92 |
| 0949 | Endocrine disease, other | T99 |
| 1109 | Anaemia, *other deficiency* | B81 |
| 1221 | Lymphadenitis, chronic, *not specified* | B71 |
| 1260 + 1341 + 1342 | Depressive disorder | P76 |
| 1280 | Organic psychosis | P71 |
| 1290 | Psychosis | P98 |
| 1311 | Anorexia nervosa | T06 |
| 1312 | Somatoform disorder | P75 |
| 1321 | Phobia | P79.01 |
| 1322 | Anxiety disorder | P74 |
| 1330 | Obsessive-compulsive disorder | P79.02 |
| 1351 | Irritable bowel syndrome | D93 |
| 1359 | (Chronic) functional somatic symptoms^†^ | P01, P78 |
| 1580 | Epilepsy | N88 |
| 1590 | Migraine | N89 |
| 1790 | Cataract | F92 |
| 1849 | Chronic otitis media | H74.01 |
| 1860 | Meniere disease | H82.01 |
| 2072 | Restless legs syndrome | N04 |
| 2100 | Rheumatic fever | K71.01 |
| 2232 | Pulmonary embolism | K93 |
| 2239 | Peripheral arterial disease; Raynaud’s disease | K99 |
| 2240 | Varicose veins; venous insufficiency | K95, K99.04 |
| 2280 | Varicose ulcer | S97.01 |
| 2472 | Bronchitis | R78 |
| 2500 | Chronic sinusitis | R75.02 |
| 2764 | Oesophageal disease | D84 |
| 2770 | Stomach ulcer | D86.01 |
| 2780 | Duodenal ulcer | D85 |
| 2790 | Peptic ulcer, *other* | D86 |
| 2841 | Diaphragmatic hernia | D90 |
| 2851 | Colonic diverticula; diverticulitis | D92 |
| 2884 | Pancreatic disease; other | D99 |
| 3101 | Glomerulonephritis | U88 |
| 3102 | Glomerulonephrosis | U88 |
| 3120 | Urinary calculi / urinary tract stones | U95 |
| 3140 | Urinary tract infection, chronic / recurrent | U71 |
| 3180 | Prostatic hyperplasia / hypertrophy | Y85 |
| 3390 | Urinary incontinence | U04 |
| 3722 | Hidradenitis | S92.02 |
| 3780 | Seborrhoeic dermatitis | S86 |
| 3790 | Atopic dermatitis | S87 |
| 3801 | Contact dermatitis | S88.01 |
| 3900 | Chronic skin ulcer | S97 |
| 4152 | Polymyalgia rheumatica; giant cell arteritis | K99.05, L99.12 |
| 4170 | Autoimmune diseases | K99, L99, S99, U99 |
| 4320 | Hydrocephalus | N85.02 |
| 4330 | Congenital cardiovascular anomaly | K73 |
| 4340 | Cleft palate | D81.01 |

*Note: For conditionally chronic diseases, only episodes assigned “ongoing attention” are counted as chronic disease.

^†^Chronic functional somatic symptoms are chronic symptoms in patients who experience functional impairment and for which a medical (organic) explanation cannot be found. GPs in the Continuous Morbidity Registration tended to classify patients as such after three episodes of presenting with functional somatic symptoms.^35^
