# Supplementary material for: Prevalence and incidence density rates of chronic comorbidity in type 2 diabetes patients: an exploratory cohort study
Source: BMC Med. 2012 Oct 29;10:128. doi: 10.1186/1741-7015-10-128 (PMC3523042; doi:10.1186/1741-7015-10-128)
Supplement: Additional file 2 — Clusters of comorbidity. [file 1741-7015-10-128-S2.DOC]

**Additional file 2: Clusters of comorbidity**

Chronic comorbid diseases classified into clusters*

| **Cluster of chronic diseases** | **Disease** |
| --- | --- |
| Cardiovascular | TIA (transient ischemic attack) |
|  | CVA (cerebrovascular accident) |
|  | Heart valve disease |
|  | Myocardial infarction |
|  | Angina pectoris |
|  | (Congestive) heart failure / heart decompensation |
|  | Atrial fibrillation / flutter |
|  | Hypertension |
|  | Intermittent claudication |
|  | Peripheral arterial disease; Raynaud’s disease^†^ |
|  | Varicose veins / venous insufficiency^†^ |
|  | Congenital heart defects^†^ |
| Musculoskeletal | Rheumatoid arthritis; ankylosing spondylarthritis |
|  | Osteoarthritis, hip |
|  | Osteoarthritis, knee |
|  | Lumbar osteoarthritis |
|  | Osteoarthritis, cervical spine |
|  | Osteoarthritis, other |
|  | Polymyalgia rheumatica; giant cell arteritis^†^ |
|  | Osteoporosis |
| Mental  Mental *(continued)* | Schizophrenia |
|  | Depressive disorder^†^ |
|  | Alzheimer’s disease |
|  | Organic psychosis^†^ |
|  | Psychosis^†^ |
|  | Phobia^†^ |
|  | Anxiety disorder^†^ |
|  | Obsessive-compulsive disorder^†^ |
|  | (Chronic) functional somatic symptoms^†^ |
|  | Personality disorder |
|  | Mental retardation |
| Eye & Ear | Cataract^†^ |
|  | Glaucoma |
|  | Blindness / amblyopia |
|  | Chronic otitis media^†^ |
|  | Meniere disease^†^ |
|  | Otosclerosis |
|  | Deafness |
| (Male and female) urogenital | Uterine fibroid / uterine leiomyoma^†^ |
|  | Glomerulonephritis^†^ |
|  | Glomerulonephrosis^†^ |
|  | Urinary calculi / urinary tract stones^†^ |
|  | Urinary tract infection, chronic / recurrent^†^ |
|  | Prostatic hyperplasia / hypertrophy^†^ |
|  | Urinary incontinence^†^ |
| Respiratory | Sarcoidosis^†^ |
|  | Asthma^†^ |
|  | COPD (chronic obstructive pulmonary disease) |
|  | Chronic sinusitis^†^ |
|  | Pneumoconiosis |
|  | Bronchiectasis |
| Skin  Skin *(continued)* | Hidradenitis^†^ |
|  | Seborrhoeic dermatitis^†^ |
|  | Atopic dermatitis^†^ |
|  | Contact dermatitis^†^ |
|  | Psoriasis |
|  | Chronic skin ulcer^†^ |
| Digestive | Irritable bowel syndrome^†^ |
|  | Oesophageal disease^†^ |
|  | Stomach ulcer^†^ |
|  | Duodenal ulcer^†^ |
|  | Diaphragmatic hernia^†^ |
|  | Colonic diverticula; diverticulitis^†^ |
|  | Crohn’s disease; ulcerative colitis |
|  | Hepatic cirrhosis |
|  | Pancreatic disease; other^†^ |
|  | Cleft palate^†^ |
| Endocrine and metabolic | Hyperthyroidism^†^ |
|  | Hypothyroidism |
|  | Gout^†^ |
|  | Endocrine disease, other^†^ |
| Neurological | MS (multiple sclerosis) |
|  | Parkinson’s disease |
|  | Epilepsy^†^ |
|  | Migraine^†^ |
| Blood(forming organs) and lymphatics | Pernicious anaemia |
|  | Anaemia, *other deficiency*^†^ |
| General and unspecified | Down syndrome / other specified congenital abnormalities |
| Infectious | Pulmonary tuberculosis^†^ |
|  | Syphilis^†^ |
| Malignancies  Malignancies *(continued)* | Cancer of the mouth / pharynx |
|  | Oesophageal cancer |
|  | Cancer of the stomach |
|  | Colon cancer |
|  | Rectal cancer |
|  | Pancreatic cancer |
|  | Laryngeal / throat cancer |
|  | Lung / bronchial cancer |
|  | Breast cancer |
|  | Uterine cervical cancer |
|  | Endometrial cancer |
|  | Prostate cancer |
|  | Bladder cancer |
|  | Ovarian cancer |
|  | Genitourinary cancer, other |
|  | Skin cancer |
|  | Brain cancer / tumour |
|  | Leukaemia |
|  | Lymphoma / multiple myeloma |
|  | Metastases; unknown origin |
|  | Carcinoma, other |

*Note: Only chronic comorbid diseases occurring at least once in the study population were classified into clusters.

^†^Conditionally chronic disease, requiring physician-assigned “ongoing episodes” at the patient level in order to be assigned as chronic disease.
